# S02 Physical activity surveillance across the life-course: from data to policy

**DOI:** 10.1093/eurpub/ckac093.005

**Published:** 2022-08-29

**Authors:** Stephen Whiting, Julianne Williams, Romeu Mendes, Sanja Music Milanovic, Viktoria A Kovacs, Kremlin Wickramasinghe

**Affiliations:** 1 Division of Country Health Programmes, WHO Regional Office for Europe, Copenhagen, Denmark; 2 EPIUnit – Instituto de Saúde Pública, Universidade do Porto, Porto, Portugal; 3 Croatian Institute of Public Health, Zagreb, Croatia; 4 School of Medicine, University of Zagreb, Zagreb, Croatia; 5 Hungarian School Sport Federation, Budapest, Hungary

**Keywords:** Physical activity, surveillance, policy, noncommunicable diseases

## Abstract

As a major risk factor for non-communicable diseases (NCDs), physical inactivity is a prominent challenge for public health. The purpose of this symposium is to present an overview of physical inactivity prevalence in Europe using data collected through international surveillance initiatives, while highlighting successes and discussing future challenges for utilising data to promote physical activity across the life course.

The World Health Organization (WHO) is involved in several physical activity surveillance systems on which discussions during this symposium will be based:

1. The WHO European Childhood Obesity Surveillance Initiative (COSI) is the largest survey of its kind in the world. An overview of results from the most recent round of COSI will be presented focusing on prevalence estimates for physical activity, screen time and sleep. Variations between countries of the socioeconomic determinants of physical inactivity, as well as the impact of the COVID-19 pandemic, will demonstrate the ongoing need for quality data to guide targeted policy actions in support of vulnerable groups throughout the life course.

2. The WHO’s STEPwise approach to surveillance (STEPS) survey provides important national level prevalence data on the behavioural and metabolic risk factors for NCDs. For many countries, the STEPS survey has provided the first estimates of physical inactivity and sedentary behaviour in the population and has been essential in raising awareness of the need for national policy actions to increase population levels of physical activity.

3. The European Commission, with the support of the WHO Regional Office for Europe, has established the European Union Physical Activity Focal Points Network to monitor implementation of the 23 indicators of the Health-Enhancing Physical Activity (HEPA) monitoring framework on physical activity policy development and implementation.

4. Within the European Union Physical Activity Focal Points Network, and during the COVID-19 pandemic, some countries established a survey on physical activity and screen time in children and adolescents.

